# Food allergy: What are people looking for? An infodemiology study

**DOI:** 10.1002/clt2.12322

**Published:** 2023-12-20

**Authors:** Karla Robles‐Velasco, Matias Panchana‐Lascano, Flavio Veintemilla‐Burgos, Romina Hinostroza, Jonathan A. Bernstein, Ivan Cherrez‐Ojeda

**Affiliations:** ^1^ Universidad Espiritu Santo Samborondon Ecuador; ^2^ Respiralab Research Group Guayaquil Ecuador; ^3^ Universidad Catolica Santiago de Guayaquil Guayaquil Ecuador; ^4^ Division of Immunology, Allergy Section Department of Internal Medicine University of Cincinnati College of Medicine Cincinnati Ohio USA

To the Editor

Food allergy (FA), an immunoglobulin E (IgE) reaction, is rising steadily over time.^1^ FA is present in 10% of the population, varying according to region.^2^ Google's search engine has become a significant source of medical information; however, this information varies in quality. Google Trends (GTr) is a free online service that gives users access to current and historical data on Google searches from 2004 up to the present.^3^

This study aims to assess the public interest and information‐seeking behavior regarding food allergies over a specified period. We conducted several searches on GTr (http://trends.google.com) on April 1st, 2023. The data was downloaded and compiled at once across 10 countries from January 1, 2012, to December 31, 2022. Based on a literature review indicating high prevalence in various countries three different types of food were selected as the primary allergens.^4^ These allergens were used as search topics in GTr, and include: “milk allergy,” “peanut allergy,” and “shellfish allergy.” The countries were selected using GTr performing a search for “Food allergy” as a topic with the "worldwide" category, we analyzed the countries with a relative search volume (RSV) score higher than 50. Ten countries were included: Australia, Canada, Finland, Hungary, Ireland, Netherlands, New Zealand, Philippines, Singapore, and the USA. Ten different searches were performed, one search for each of the 10 countries. Each search containing the three allergens, and done under the category “Health” in GTr. RSV is a value that expresses the relation between a specific search concerning the overall searches on Google at a specific time among other modifiable variables. RSV ranges between 100 and 0 in descending order, the former being a high popularity and the latter a low one. For this study, search volume trends and interest over time were developed using Statistical Package for Social Science (version 21.0; SPSS).

## 1 INTEREST OVER TIME

**FIGURE 1:**
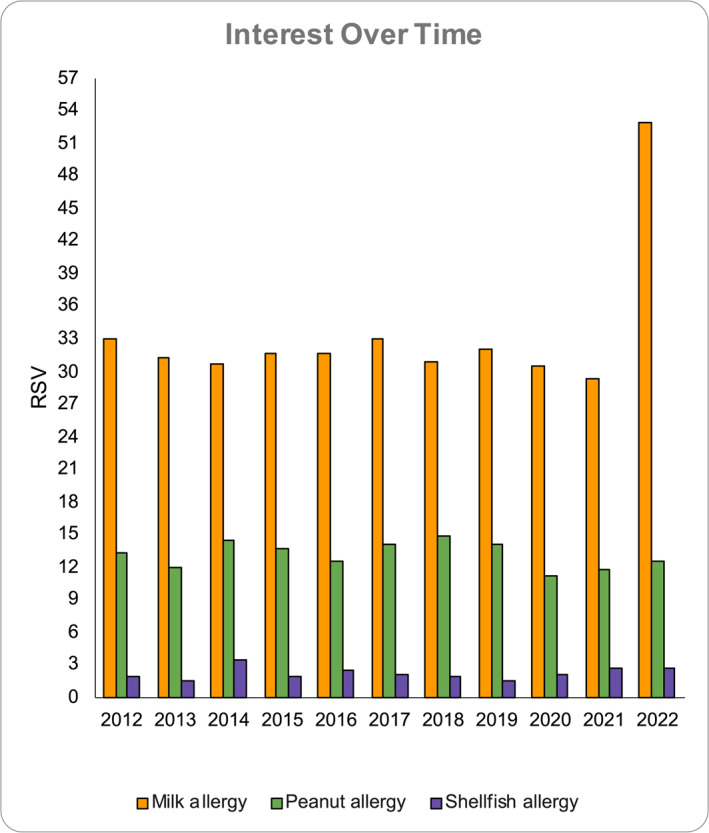
Interest over time of different allergens from 2012 to 2022.

Over the years, there has been a noticeable growth in people's interest in searching about “food allergy” as a topic of search, as evidenced by increased Google search trends from 2012 to 2022 in the 10 countries examined. Although the interest in peanut allergy has been relatively low in the last decade, in the last 2 years there has been a prominent increase in searches in Australia, the USA, Finland, the Netherlands, and New Zealand. On the other hand, there was a decreasing interest in Canada, Hungary, Ireland, and the Philippines. Milk allergy was the most searched topic in 6 out of 10 countries of interest, with a sharp spike since 2021 in Canada, the USA, and Australia. In contrast, “shellfish allergy” showed the lowest RSV, being only searched mainly in the Philippines and Singapore (Figure 1).

## 2 MOST SEARCHED TOPICS

The related queries with the most interest were different terms and definitions for allergy, such as “allergy,” “allergies,” “food allergy,” and “peanut allergy.” Among the most searched signs and symptoms were “rash,” “eczema,” “urticaria,” and “anaphylaxis.” The country with the highest searches for signs and symptoms was the US, and the allergen was shellfish. There was a high interest in searching for some related diseases to milk allergy, including “lactose intolerance,” “dairy intolerance” and “celiac disease.” The countries that most sought diagnostic methods were Ireland and Hungary, with queries such as “food allergy test” and “food intolerance testing.” “Baby” and “toddler” were the age‐related searches, the first being included in the top five searched entities in eight out of 10 countries. “Epinephrine,” “immunoglobulin,” “cetirizine,” and “antihistamine” were identified within the therapeutic queries with the highest RSV in the Philippines (Table 1).

**TABLE 1:** Average RSV of related queries from all 10 countries, divided in seven categories.

Allergy terms		Related diseases		Clinical manifestations		Affected population		Diagnostic tools		Treatment		Others	
Allergy	100	Lactose intolerance	100	Hives	100	Baby allergy	44	Allergy testing	41	Cetirizine	100	Peanut oil	6
Peanut allergy	100	Celiac disease	55	Lactose intolerance symptoms	41	cow's milk allergy baby	30	Food allergy testing	40	Antihistamine	84	Dog food allergies	4
Milk allergy	100	Food intolerance	16	Seafood allergy symptoms	34	Toddler	20	Allergy testing Dublin	10	Allergy treatment	9	Dog allergies	4
Food allergy	100	Intolerance	13	Symptoms of milk allergy	34	Milk allergy baby	17	Food allergy test	9	Food allergy treatment	9	Food allergy cat	4
Seafood allergy	98	Milk intolerance	12	Anaphylaxis	30	Little boy allergy	14	Food allergy testing Dublin	9	Peanut allergy treatment	4	Pepti	3
Dairy	60	Dairy intolerance	7	Milk allergy symptoms	23	Adult food allergy	12	Allergy test	8	Peanut allergy cure	4	Nutrilon	2
Dairy allergy	59	Cereal allergy	3	Food allergy symptoms	15	Baby dairy allergy	7	Food intolerance testing	6	Epipen	3	g6pd allergy	2
Allergie alimentaire	19	Cross allergy	3	Dairy allergy symptoms	15	Baby food allergy	5	Food allergy testing Ireland	4	Peanut allergy cure	2	Gastroenterology	2
Allergy to milk	17	Wheat allergy	3	Allergy rash	15	Baby formula	4	Food allergy test Budapest	3	Milk‐free diet	2	Anaphylaxis is caused by what body system?	2
Skin allergy	11	Grain allergy	3	Food allergy rash	15	Baby peanut allergy	3	Food intolerance test	2	Lactose free	2	Foodborne illness is an illness caused by?	2
Allergic	11	Lactose intolerant	2	Allergy symptoms	14			Synlab	2			Peanut allergy vaccine	1
Food allergy is	11	Soy allergy	2	Food allergy symptoms	14							I suffer from a seafood allergy	1
Cows milk allergy	11	Soy milk	2	Milk allergy rash	10							A customer with a peanut allergy wants to buy some biscuits and asks if your biscuits contain nuts. The best response is	1
Allergic to peanuts	9	Milk sugar sensitivity	2	Seafood allergy rash	8								
Shellfish allergy	7	Food poisoning	1	Seafood allergy reaction	7								
Common food allergies	6	Celiac	1	Allergic reaction	4								
Common allergies	6			Shrimp allergy	4								
What is a food allergy	6			Shellfish allergy symptoms	3								
Fish allergy	4			Fish allergy symptoms	2								
Shrimp allergy	4			Shrimp allergy symptoms	2								
Soy allergy	4			Eczema	2								
Allergy to peanuts	3			Cow's milk allergy symptoms	2								
Peanut oil peanut allergy	3			Symptoms cow's milk allergy	2								
Nut allergy	3												

Abbreviation: RSV, related search volume.

Google is a tool for gathering information.^3^ With the birth of GTr, researchers in the field of infodemiology have been using this tool to analyze the population's behavior. In the medical field, some examples are forecasting epidemiological traits of allergic rhinitis, establishing correlation between asthma hospitalizations and common cold, revealing spikes of interest in nasal polyps, among many others.^5^, ^6^, ^7^ The spike of interest in milk allergy in 2021 for 6 out of the 10 selected countries might be related to the findings of a study in the UK^8^ which showed that cow's milk allergy may be the root of three‐quarters of newborns' two or more allergic symptoms at some point in the first year of life. Another possible reason for the trending phenomenon may be the hospitalizations and deaths of infants in the USA because of contaminated Abott baby formula products in May of 2022.^9^ The worldwide prevalence of shellfish allergy ranges from 0.2% to 0.6%, but this number is much higher in the Asia‐Pacific region.^10^ Within this area, teenagers in the Philippines and Singapore report the highest prevalence of this allergy, with 5.12% and 5.13% respectively.^10^ This data correlates with our findings. Food allergy, particularly IgE‐mediated, can present with clinical manifestations in different systems.^11^ “Hive,” “rash,” “eczema,” and “anaphylaxis” were the most searched clinical signs in our study. This shows that users have a concern for cutaneous manifestations and life‐threatening ones. The current management of FA relies on the avoidance of allergens, preparedness to promptly address allergic reactions, and interventions to alleviate symptoms, including the administration of antihistamines and immunotherapy.^11^ We found a considerable level of interest in the 10 chosen countries regarding food alternatives such as “almond milk” and “soy milk,” as well as “epinephrine” due to its critical role as the primary therapeutic intervention for anaphylaxis, an acute and potentially life‐threatening allergic reaction.^12^

## 3 CONCLUSION

The findings of this study show how GTr is a powerful tool that can be used to determine the user's interest in FA and how this interest can fluctuate. This study is the first of its kind, analyzing data from what people search in the web regarding FA. Similarly to other infodemiology studies, by incorporating real‐world data in a nationwide perspective, its findings may be useful for policymakers or guideline developers. Making public health information interesting and engaging, is essential for effective communication and overall success of health initiatives.

## AUTHOR CONTRIBUTIONS

**Karla Robles‐Velasco**: Conceptualization (equal); data curation (equal); formal analysis (equal); funding acquisition (equal); investigation (equal); methodology (equal); project administration (equal); resources (equal); supervision (equal); validation (equal); visualization (equal); writing – original draft (equal); writing – review & editing (equal). **Matias Panchana‐Lascano**: Conceptualization (equal); data curation (equal); formal analysis (equal); investigation (equal); methodology (equal); project administration (equal); resources (equal); software (equal); writing – original draft (equal); writing – review & editing (equal). **Flavio Veintemilla‐Burgos**: Conceptualization (equal); data curation (equal); formal analysis (equal); investigation (equal); methodology (equal); project administration (equal); writing – original draft (equal); writing – review & editing (equal). **Romina Hinostroza**: Conceptualization (equal); data curation (equal); formal analysis (equal); investigation (equal); methodology (equal); visualization (equal); writing – original draft (equal); writing – review & editing (equal). **Jonathan A. Bernstein**: Conceptualization (equal); data curation (equal); formal analysis (equal); investigation (equal); methodology (equal); project administration (equal); validation (equal); visualization (equal); writing – original draft (equal); writing – review & editing (equal). **Ivan Cherrez‐Ojeda**: Conceptualization (equal); data curation (equal); formal analysis (equal); funding acquisition (equal); investigation (equal); methodology (equal); project administration (equal); resources (equal); software (equal); supervision (equal); validation (equal); visualization (equal); writing – original draft (equal); writing – review & editing (equal).

## CONFLICT OF INTEREST STATEMENT

The authors declare that there is no conflict of interest regarding the publication of this paper.

## FUNDING INFORMATION

Universidad Espiritu Santo, Grant/Award Number:2023‐MED‐008

## Data Availability

Data available on request from the authors.
